# Association of Perinatal Cardiovascular Features with Angiotensin System Expressions in Maternal Preeclampsia

**DOI:** 10.3390/ijms25137426

**Published:** 2024-07-06

**Authors:** I-Chun Lin, Kay L. H. Wu, Hsin-Hsin Cheng, Ching-Chang Tsai, Hong-Ren Yu, Te-Yao Hsu, You-Lin Tain, Li-Tung Huang, Yun-Ju Lai

**Affiliations:** 1 Department of Pediatrics, Kaohsiung Chang Gung Memorial Hospital and Chang Gung University, College of Medicine, Kaohsiung 833, Taiwan; yuu2002@cgmh.org.tw (H.-R.Y.); tainyl@cgmh.org.tw (Y.-L.T.); litung.huang@gmail.com (L.-T.H.); 2 Institute for Translational Research in Biomedicine, Kaohsiung Chang Gung Memorial Hospital and Chang Gung University, College of Medicine, Kaohsiung 833, Taiwan; klhwu@cgmh.org.tw; 3 Department of Obstetrics and Gynecology, Kaohsiung Chang Gung Memorial Hospital and Chang Gung University, College of Medicine, Kaohsiung 833, Taiwan; chokovarous@cgmh.org.tw (H.-H.C.); aniki@cgmh.org.tw (C.-C.T.); tyhsu@cgmh.org.tw (T.-Y.H.)

**Keywords:** aminopeptidase-N, angiotensin II receptors, blood pressure, cardiovascular, coronary artery, developmental origin of health and disease, neonate, offspring, perinatal, preeclampsia

## Abstract

We hypothesized and investigated whether prenatal exposure to preeclampsia (PE) would simultaneously affect perinatal cardiovascular features and angiotensin system expressions. This prospective study was composed of mother-neonate dyads with (n = 49) and without maternal preeclampsia (n = 48) in a single tertiary medical center. The neonates exposed to PE had significantly larger relative sizes for the left and right coronary arteries and a higher cord plasma level of aminopeptidase-N, which positively correlated with the maternal diastolic blood pressures and determined the relative sizes of the left and right coronary arteries, whereas the encoding aminopeptidase-N (*ANPEP*) mRNA level in the PE cord blood leukocytes was significantly decreased, positively correlated with the neonatal systolic blood pressures (SBPs), and negatively correlated with the cord plasma-induced endothelial vascular cell adhesion molecule-1 mRNA levels. The PE cord plasma significantly induced higher endothelial mRNA levels of angiotensin II type 1 receptor (*AT1R)* and *AT4R,* whereas in the umbilical arteries, the protein expressions of AT2R and AT4R were significantly decreased in the PE group. The endothelial *AT1R* mRNA level positively determined the maternal SBPs, and the *AT4R* mRNA level positively determined the neonatal chamber size and cardiac output. In conclusion, PE may influence perinatal angiotensin system and cardiovascular manifestations of neonates across placentae. Intriguing correlations between these two warrant further mechanistic investigation.

## 1. Introduction

Preeclampsia (PE) is a one of the most common causes of mortality and morbidity for both pregnant women and neonates [1,2], resulting from abnormal placentation and leading to impaired uterine arterial flow and placenta insufficiency. It is noteworthy that cumulative evidence shows the importance of the developmental origins of health and disease (DoHaD) in PE [3], which indicates that the offspring of maternal PE have increased rates of hypertension and coronary heart disease [4,5]. Some studies showed that PE begins impacting the offspring’s cardiovascular system in the fetal stage [6] and persists from the antenatal to postnatal stages [7]. Indeed, we recently found that maternal PE may lead to coronary inflammation and affect the cardiovascular features of neonatal offspring at birth [8].

Dysregulation of the angiotensin system is associated with an imbalance of vasodilation and vasoconstriction, and it may be involved in the pathogenesis of PE [9]. Blood pressure is regulated by the renin–angiotensin system, which involves several steps of conversion from angiotensin I (AngI) to AngII, Ang1–9, Ang1–7, AngIII, and then AngIV through catalytic metabolism with angiotensin converting enzyme (ACE), ACE2, neprilysin (NEP), aminopeptidase A, and aminopeptidase-N (APN). The binding of AngII and AngIII to angiotensin II type I receptor (AT1R) on the vasculature leads to vasoconstriction, whereas the binding of AngII to AT2R and Ang1–7 to MAS1 receptor on the vasculature leads to vasodilatation [10]. However, whether the PE alters the angiotensin system in the neonates and whether such alterations impact the cardiovascular manifestations in the PE offspring remains discovered. Therefore, we aimed to test our hypothesis in this study and found that certain cell- and tissue-specific expressions of maternal and neonatal angiotensin-related molecules (e.g., APN, AT1R, and AT2R) were altered in the PE group. Some of these alterations could determine the perinatal cardiovascular features (e.g., systolic blood pressure and cardiac function). This study ignites the drive to explore the mechanistic impact of PE on cardiovascular health in offspring.

## 2. Results

### 2.1. Cardiovascular Features in the Studied Groups

The characteristics of the study population, comprising 48 pairs of mothers and neonates in each group and one twin in the PE group, are shown in the Table 1. Of the PE mothers, approximately 85% had PE with severe features, 8% had prior essential hypertension, and 96% had proteinuria. None of the PE mothers exhibited eclampsia. The median gestational age (GA) of initially presented or diagnosed PE was 27 (IQR: 21.50–31.25) weeks. Most of the neonatal cardiovascular measurements, including the relative sizes of the left main coronary artery (LCA) and right coronary artery (RCA) (the ratios of LCA and RCA over the aorta) [8], were significantly lower in the PE group than those in the normotension group, except for the heart rate, absolute inner diameters of the LCA and RCA, and the fractional shortening, ejection fraction (EF), and cardiac index (CI) of the left ventricle (LV). Owing to the significant difference in GA between the two groups, a comparison was further performed using binary logistic regression in the model of GA adjustment. Most of these cardiovascular features became insignificantly different, but the absolute LCA diameter (odds ratio: 218.09; 95% C.I., 2.76–17,236.09; *p* = 0.016) and the relative LCA and RCA sizes (*p* = 0.001 and *p* = 0.022, respectively) remained significantly larger in the PE group.

### 2.2. Expressions and Correlations of Angiotensin-Related Molecules to Perinatal Cardiovascular Features

**Figure 1:**
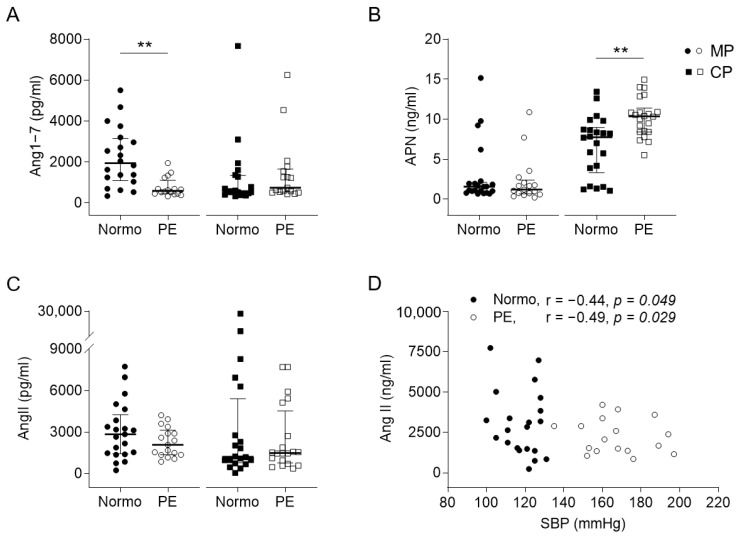
The concentrations of angiotensin 1–7 (Ang1–7), aminopeptidase-N (APN), and angiotensin II (AngII) in the plasma from maternal and cord bloods. (**A**–**C**) The maternal (MP) and cord plasma (CP) levels of Ang1–7, APN, and AngII in the normotension (Normo) and preeclampsia (PE) groups. (**D**) The correlations of maternal systolic blood pressure (SBP) with the MP level of AngII. Horizontal lines on scatter dot plots indicate median with IQRs (n = 20~22 in control; n = 17~20 in PE) ** *p* < 0.01.

**Figure 2:**
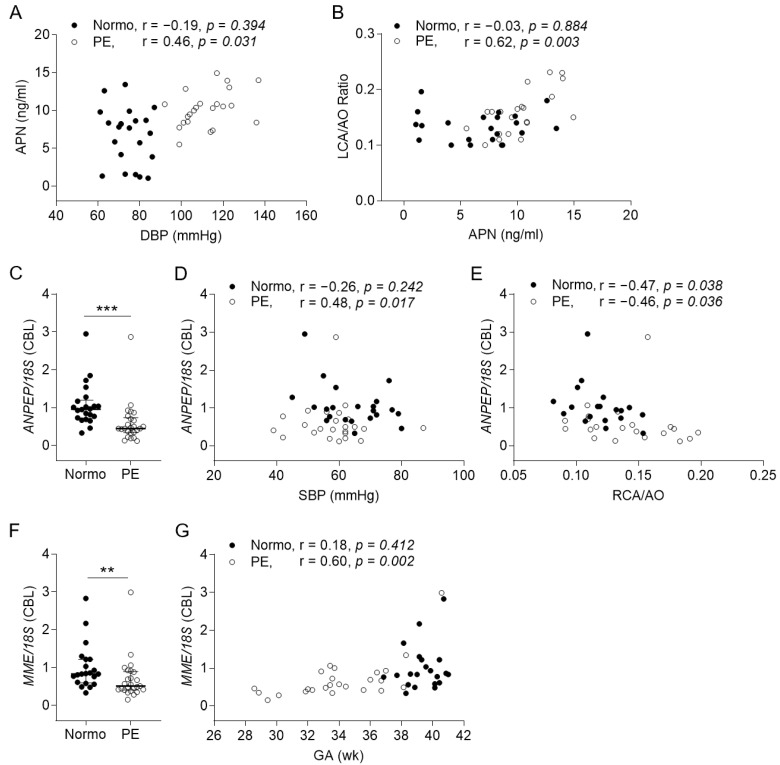
Correlations of the cord plasma APN level and the *ANPEP* and *MME* mRNA levels in cord blood leukocytes (CBLs) with cardiovascular features or gestational ages (GAs). (**A**,**B**) The correlations of the CP concentrations of APN with the antepartum maternal diastolic blood pressure (DBP) and the ratios of the left main coronary artery (LCA) to the aorta (AO). (**C**,**F**) The relative mRNA levels of the *ANPEP* and *MME* genes in the CBLs. (**D**,**E**) The correlations of the relative *ANPEP* mRNA expression levels in the CBLs with the neonatal SBP and relative sizes of the right coronary artery (RCA; the ratios of RCA to AO). (**G**) The correlations of the relative *MME* mRNA expression levels in the CBLs with GAs. Horizontal lines on scatter dot plots indicate median with IQRs (n = 20~22 in control; n = 25 in PE). ** *p* < 0.01. *** *p* < 0.001.

Next, we compared the expression levels of the angiotensin-related molecules in maternal cord bloods between the two groups (Figure 1). The PE group had significantly lower maternal plasma (MP) levels of Ang1–7 and a higher cord plasma (CP) level of APN than the control. Although the MP level of AngII was not significantly different between the two groups, both of them were negatively correlated with the maternal systolic blood pressure (SBP) (Figure 1C,D). Furthermore, the CP level of APN was positively correlated with the maternal diastolic blood pressure (DBP) and the relative size of the neonatal LCA in the PE group (Figure 2A,B). The *ANPEP* and *MEM* mRNA expression levels of the cord blood leukocytes (CBLs) in the PE group were significantly lower than those in the normotension group (Figure 2C,F). The former was positively correlated with the neonatal SBP and negatively correlated with the neonatal relative RCA size (Figure 2D,E). The latter was positively correlated with the neonatal GA (Figure 2G). Otherwise, the CP levels of Ang1–7, the MP level of APN, the levels of AngII and NEP in both MP and CP, the mRNA expression levels of the *AT1R*, *AT2R*, *AT4R*, *MAS1*, *ACE*, and *ACE2* genes in both the maternal blood leukocytes (MBLs) and CBLs, and the mRNA expression levels of *ANPEP* and *MME* in the MBLs were not significantly different between the two groups (Supplementary Table S1).

### 2.3. In Vitro Effect of MP and CP on Endothelial Cells

**Figure 3:**
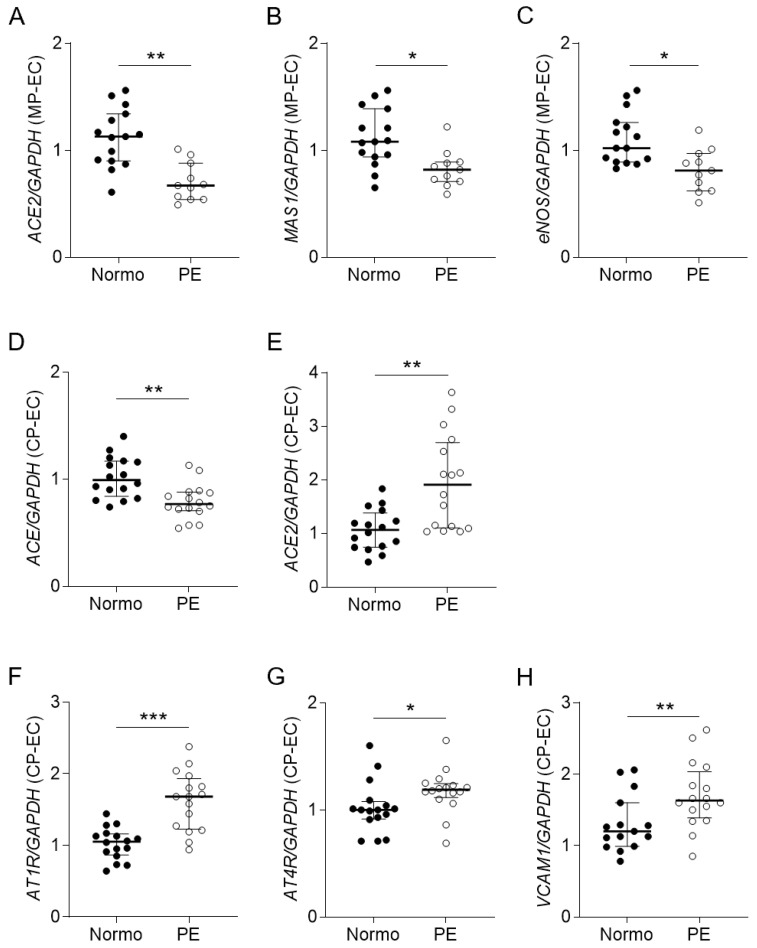
The mRNA expressions of the MP- and CP-stimulated endothelial responses. (**A**–**C**) The relative mRNA expression levels of angiotensin converting enzyme 2 (*ACE2*), *MAS1*, and endothelial nitric oxide synthase (*eNOS*) genes in the MP-stimulated human umbilical artery endothelial cells (HUAECs; MP-EC) (n = 12 in control; n = 11 in PE). (**D**–**H**) The relative mRNA expression levels of *ACE*, *ACE2*, angiotensin II type 1 (*AT1R*) and 4 receptors (*AT4R*), and vascular cell adhesion molecule-1 (*VCAM1*) genes in the CP-stimulated HUAECs (CP-EC) (n = 16 each group). Horizontal lines on scatter dot plots indicate median with IQRs. * *p* < 0.05. ** *p* < 0.01. *** *p* < 0.001.

**Figure 4:**
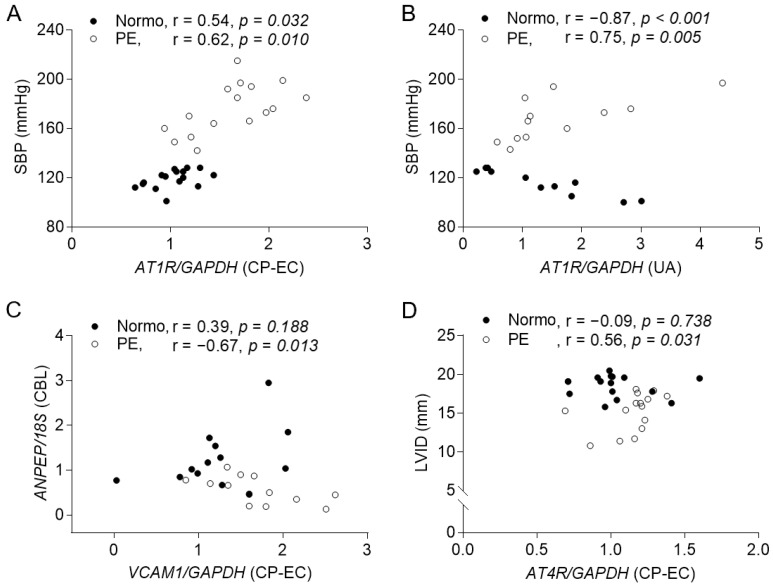
The correlations of the relative mRNA expression levels with the cardiovascular features**.** (**A**,**B**) The correlations of the relative *AT1R* mRNA expression levels in CP-stimulated HUAECs and in umbilical arteries with maternal SBP. (**C**) The correlations of the CP-induced endothelial *VCAM 1* mRNA expression level with the relative *ANPEP* mRNA expression level in CBLs. (**D**) The correlation of the CP-induced endothelial *AT4R* mRNA expression level with the neonatal LV chamber size.

While examining the influence of PE plasma on the endothelium, we found that the MP-stimulated human umbilical artery endothelial cells (HUAECs) significantly produced lower mRNA levels of *ACE2*, *MAS1*, and *eNOS*, and the CP-stimulated HUAECs produced a significantly lower mRNA level of *ACE* but higher levels of *AT1R*, *AT4R*, *ACE2*, and vascular cell adhesion molecule (*VCAM*)-1 in the PE group (Figure 3). Additionally, the CP-induced endothelial and umbilical arterial *AT1R* mRNA levels were significantly correlated with the maternal SBPs in both groups (Figure 4A,B), although the umbilical arterial *AT1R* mRNA level was not significantly different between the two groups. In the PE group, the CP-induced endothelial *VCAM1* mRNA level was negatively correlated with the *ANPEP* mRNA level in the CBLs, and the endothelial *AT4R* mRNA level was positively correlated with the neonatal inner dimension of the LV (LVID) (Figure 4C,D).

### 2.4. Local Expression of Angiotensin Receptors and Function of Umbilical Artery

**Figure 5:**
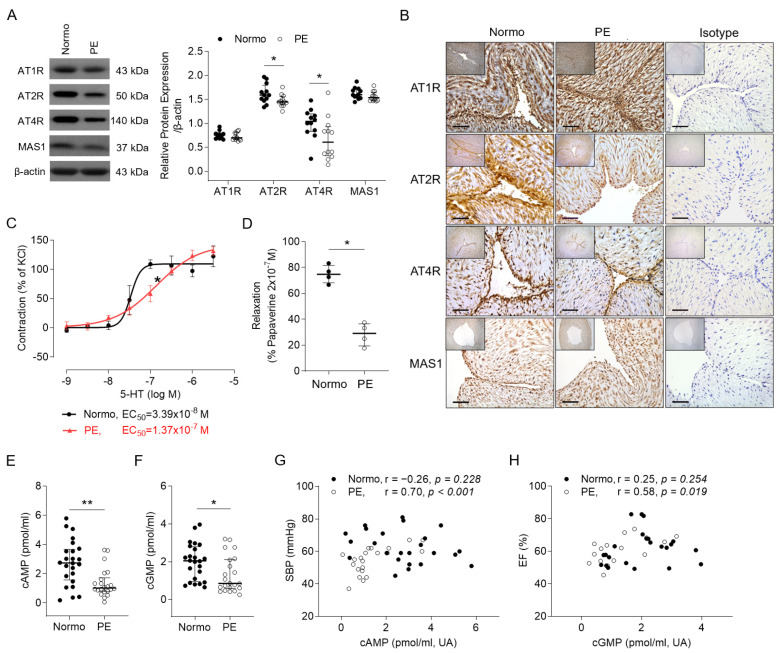
The expressions of angiotensin-related molecules and the functions of the umbilical arteries (UAs). (**A**) Representative western blots and quantitative analysis of protein expression levels of AT1R, AT2R, AT4R, and MAS-1 (n = 12 each group). (**B**) Representative microphotographs of immunochemical staining showing decreases in AT2R and AT4R protein expression over the endothelium and vascular cells of the UAs in the PE group (original magnification of 400× and 100× in left upper boxes; bar = 20 μm). (**C**,**D**) The functions of vasoconstriction and vasorelaxation of the UAs were depressed in the PE group. (**E**,**F**) The concentrations of cyclic adenosine monophosphate (cAMP) and cyclic guanosine monophosphate (cGMP) in the UAs. (**G**) The correlations of umbilical arterial levels of cAMP with the neonatal SBP. (**H**) The correlations of umbilical arterial levels of cGMP with the neonatal ejection fraction (EF) of the left ventricle. Values are expressed as the mean ± standard error in C (n = 4 each group). Horizontal lines on dot plots indicate median with IQRs. * *p* < 0.05. ** *p* < 0.01. *5-HT* = serotonin hydrochloride.

In the local vascular tissue, the umbilical arteries (UAs) exhibited significantly lower protein expression levels for AT2R and AT4R in the PE group (Figure 5A). Although the PE group had a significantly higher arterial *ACE* mRNA level (median relative expression, 1.62-fold; IQR 1.15–2.10-fold versus the median, 1.12-fold; IQR 0.71–1.51-fold), there was no significant difference in the mRNA expressions of the *AT1R*, *AT2R*, *AT4R*, *MAS1*, and *ACE2* genes between groups. Meanwhile, a reduction in AT2R and AT4R staining was observed over the endothelium and within the smooth muscle cells in the UAs from the PE group through an immunohistochemistry study (Figure 5B). The vasomotor function study shows that the UAs from the PE group displayed significantly lower percentages of vascular contraction and relaxation (Figure 5C,D). The arterial concentrations of cyclic adenosine monophosphate (cAMP) and cyclic guanosine monophosphate (cGMP) in the UAs were significantly reduced in the PE group (Figure 5E,F). The former was positively correlated with the neonatal SBP, and the latter was positively correlated to the neonatal EF of the LV (Figure 5G,H).

### 2.5. Independent Factor(s) of Perinatal Cardiovascular Features under Multivariable Analysis

Finally, a multivariable analysis was performed using a stepwise linear regression to determine the cell- and tissue-specific independent factor(s) of the cardiovascular features individually for the PE and normotension groups in the GA-adjusted models (Table 2). In the PE group, the *ANPEP* mRNA level in the CBLs and arterial cAMP level could be significantly independent factors of the neonatal SBP, and the arterial *AT2R* mRNA expression level was negatively associated with the neonatal DBP. The CP-induced endothelial *AT4R* mRNA expression level positively determined the neonatal LVID and cardiac output (CO), and it was negatively correlated with the thickness of the LV’s posterior wall (LVPW). Of note, the CP level of APN could be a significant determinant of the neonatal relative LCA and RCA sizes and the end systolic volume of the LV. The CI could also be independently determined by the MP levels of Ang1–7. Additionally, the maternal SBP was positively related to the arterial and CP-induced endothelial *AT1R* mRNA expression levels, and the maternal DBP was positively related to the arterial *AT1R* mRNA expression level. In the normotension group, the arterial AT2R protein expression level positively determined the neonatal LVID, end diastolic volume (EDV), stroke volume (SV), CO, and CI, and it was negatively correlated with the neonatal DBP. The CP-induced endothelial *AT4R* mRNA level was positively correlated with the interventricular septum thickness, and the *ACE* mRNA level was negatively correlated with the EDV. The umbilical arterial *AT1R* mRNA level was negatively associated with the maternal SBP, and the arterial *ACE* mRNA level was negatively correlated with the neonatal diameter of the aortic root and the end systolic volume, EDV, and CI of the LV. The arterial cGMP level could be a significantly independent factor of the neonatal DBP, thickness of the LVPW, and SV.

## 3. Discussion

In this study, we demonstrated that PE may affect some angiotensin system expressions in both mothers and neonatal offspring and that there are some associations between these alterations and some perinatal cardiovascular features.

An imbalance between vasoconstriction and vasodilation in PE is the key consequence resulting from the complex interaction among inflammation, oxidative stress, and the angiotensin system [11,12]. Partly compatible with these, our data did show that both vasoconstriction and vasodilation of the umbilical arteries were impaired. Two of the most important regulators in the angiotensin system, AT1R and AT2R, mediate the vascular tone and conversely contribute to the development of hypertension [13]. Although it was reported that AT1R is downregulated in normal pregnancies [14], our results revealed no significant difference in umbilical arterial expression. Interestingly, the higher the maternal SBP, the lower umbilical arterial *AT1R* mRNA level in the normotension group. On the contrary, the higher the maternal SBP, the higher the arterial *AT1R* mRNA level in the PE group. Of note, the CP from the PE group could induce more *AT1R* mRNA expression along with increases in maternal SBP. This may suggest that certain substance(s) in the CP from the PE group with a higher SBP could trigger stronger signaling pathways relating to *AT1R* upregulation or dampen those relating to *AT1* downregulation. Indeed, there is a possibility that such a substance in the CP of those with severe PE or hypertension could remarkably affect the neonatal endothelium, because we found a positive association of the CP-induced *AT1R* mRNA level with the induced *VCAM1* mRNA level (Pearson’s correlation r = 0.55, *p* = 0.028). Despite little research dissecting the relationship between AT1R expression and maternal BP, this result could be practical because the AngII-AT1R axis mediates vasoconstriction, and there is a pathogenic role for increased AngII sensitivity in PE [15]. Therefore, the AT1R axis may play a potential effector role in the neonatal vasculatures, according to the severity of maternal hypertension. Until now, few publications have addressed the pathogenetic role of AT2R in human PE. Like a previous study on the placenta [16], our data show that umbilical arterial AT2R expression was depressed in the PE group. For both groups, the lower the AT2R expression, the higher the neonatal DBP. That aside, the higher the umbilical AT2R protein level, the larger the chamber size, SV, CO, and CI of the LV in the normotension group. This seems reasonable because the AngII-AT2R axis mediates vasodilatation via cGMP-dependent cascades [17], and the AT2R axis induces afterload reduction and cardiac protection [18]. Therefore, the more the AT2R axis is activated, the lower the BP and the larger the CO and CI will be. This may explain our observation regarding the association of the umbilical cGMP level with the neonatal DBP, SV, and EF.

In a normal pregnancy, the maternal circulating renin–angiotensin system shifts toward AngIV [19], although the peripheral effect of the AngIV- AT4R axis on vasodilation or vasoconstriction remains controversial [10]. Similar to previous placenta findings [20], the umbilical arterial AT4R protein expression was significantly depressed in the PE group. AT4R, also known as placental leucine aminopeptidase, which is encoded by the *LNPEP* gene, has been studied for its role in PE [19,21]. Aminopeptidases play an important role in the regulation of the systemic and local renin–angiotensin system and in the control of cardiovascular and renal function [22]. Declining activity of AT4R was observed in maternal blood in severe PE, with impaired placental blood flow [21]. Our UA finding may suggest that PE affects the fetal artery system beyond the placenta because UA originates from the internal iliac artery in babies. However, we found that the CP paradoxically induced higher endothelial *AT4R* expression in the PE group, though it was not particularly prominent. The difference between the umbilical arterial and CP-induced expressions may be partly because of in vitro endothelial responses to plasma and the entirety of in vivo arterial responses to a high-afterload placenta.

One of our striking findings is that the CP level of APN, converting AngIII to AngIV [23], independently determined the relative coronary sizes in the PE neonates. Recently, we reported that the relative coronary sizes (ratios of the LCA and RCA to the AO) might be a clinical index reflecting the effect of the PE severity on neonatal outcomes [8]. The increase in APN and the decreased *ANPEP* mRNA expression level of CBLs and their associations with the relative coronary sizes and CP-induced *VCAM1* level may suggest that the PE neonates with higher CP levels of APN or with lower *ANPEP* expression of CBLs would be those greatly affected by maternal PE and potentially at risk of unfavorable outcomes. However, further mechanistic investigation is still needed to explore the direct pathologic roles of APN, ANPEP, and AT4R on PE and their links to neonatal cardiovascular features in PE offspring.

PE and intrauterine hypoxia may be predisposing factors for fetus endothelial dysfunction [24] and oxidative stress [25]. Obviously, our data reveal that MP and CP differentially triggered endothelial responses. The MP from PE tended to attenuate the expression of vasorelaxation-related genes (e.g., *ACE2* and *MAS1*), whereas the CP from PE tended to alter both vasoconstriction- and vasodilatation-related gene expression in a more complex fashion (e.g., *AT1R* and *ACE2* upregulation and *ACE* downregulation). This may be the reason why the UAs had both impaired vasoconstriction and vasorelaxation. Of the utmost importance is that the endothelial *ACE2* expression responded differently to MP and CP stimulation. In a normal pregnancy, ACE2 expression is increased, especially in the placenta [26]. The protective role of ACE2 has been proposed in PE for counterbalancing AngII-related signaling pathways [27,28]. Future mechanistic exploration is needed to elucidate the effect of such differential ACE2 expression on PE.

In conclusion, these angiotensin-related elements were differentially altered in various cells and tissues in PE, and they may intricately cooperate to deal with health and disease. Our study results may provide convincing evidence that PE impacts the neonatal angiotensin system across the placenta and differentially influences cardiovascular manifestations of mothers and neonates via different mechanisms, which mandates further investigation and long-term follow-ups.

The limitation of this study mainly resulted from the clinical characteristics of the preterm PE neonates and the low GA. There was difficulty in recruiting healthy GA- or BBW-matched neonates as control subjects for biomaterial sampling. Since there were significant differences in age and BBW between the two groups, interpretation should be more cautious for some data with potential age-dependent expression (i.e., the lower the GA, the lower the expression of *ANPEP* mRNA in CBLs). Thus, we used a GA-adjusted model to avoid such a confounding factor, especially in the individual group. Second, some experiments, especially the in vitro cell culture and western blot, were not performed for all samples. Data interpretation and clinical implication should be more careful.

## 4. Materials and Methods

### 4.1. Subjects, Sampling, and Cardiovascular Measurements

Between July 2015 and March 2018, a prospective observational study was executed in the Departments of Pediatrics and Obstetrics at Kaohsiung Chang Gung Memorial Hospital in Taiwan. Two groups of mother-neonate dyads were recruited, including those with maternal PE (PE group), diagnosed as the guideline [29], and those without maternal systemic disease (normotension group). Preeclampsia is typically diagnosed as gestational hypertension, which develops after a GAof >20 weeks, with a SBP > 140 mmHg or DBP > 90 mmHg on two occasions at least 4 h apart, proteinuria, and generalized edema [29]. Additionally, if any of the following features are presented (SBP > 160 mmHg or DBP > 110 mmHg, thrombocytopenia, impaired liver function, renal insufficiency, pulmonary edema, new-onset headache, or visual disturbance), the severe form of PE could be diagnosed [29]. The antepartum maternal whole blood, postpartum umbilical cords, and cord blood were collected with the participants’ written permission. The SBP and DBP of the mothers and neonates were record upon admission, and neonatal cardiovascular measurements were immediately measured within 48 h with transthoracic echocardiography using a Philips IE33 or EPIQ ultrasound system (Philips Medical Systems) as previously described [8].

### 4.2. Measurements of Angiotensin-Related Molecules

Plasma and blood leukocytes were separated from the maternal or cord blood after centrifugation at 3000 rpm for 10 min. Kits for an enzyme-linked immunosorbent assay were then used to test the plasma concentrations of AngII (Enzo, Farmingdale, NY, USA), Ang1–7 (Mybiosource, San Diego, CA, USA), APN, (Bluegene, Shanghai, China), and NEP (R&D, Minneapolis, MN, USA), as well as the arterial concentrations of cAMP (Enzo) and cGMP (Enzo).

### 4.3. Cells, Tissues, and Real-Time RT-PCR

To explore the local vascular characteristics, the UAs were meticulously dissected from umbilical cords as previously described [8,30] and immediately frozen at −80 °C for further experiments. Additionally, HUAECs from the healthy group were isolated, primarily cultured, and then maintained in Medium 199 (Thermo, Waltham, MA, USA), endothelial cell growth supplement (Millipore, Burlington, MA, USA), and 10% fetal bovine serum (Thermo) at 37 °C with 5% CO_2_. For an in vitro stimulation experiment, the HUAECs were pre-seeded in 24 well plates at a density of 1 × 10^5^ cells/well overnight and then cultured in a fresh culture medium containing 15% MP or CP from a single subject for a further 24 h.

The tissues and cells were homogenized, and the total RNA was extracted from the UAs, plasma-stimulated HUAECs, MBLs and CBLs. The samples were initially added with TRIzol reagent (Thermo) to avoid RNase action and stored in a −80 °C freezer. RNase-free equipment and reagents were used in the RNA extraction process, including diethylpyrocarbonate-treated water. The RNA quality was checked using spectrophotometry (A260/A280 ratio) to ensure the samples remained uncontaminated and intact. Finally, the total RNA was reverse transcribed to cDNA as previously described [8], and real-time RT-PCR was then performed in the presence of Fast SYBR Green Master Mix using an ABI 7500 sequence detection system (Applied Biosystems, Waltham, MA, USA). The sequences of paired primers of target genes and house keeping genes are listed in Supplementary Table S2. 

### 4.4. Protein Extraction and Western Blot

Next, the total protein from umbilical arteries was homogenized and extracted with a gentleMACS™ Dissociator (Cat. 130093235, Miltenyi Biotec, Auburn, CA, USA) following the company’s protocol. In brief, each 100 mg of artery tissue was mixed with 1.5 mL of the pre-chilled PRO-PREP™ protein extract solution (Cat. 17081, iNtRON Biotechnology, Seongnam-Si, Gyeonggi-do, Republic of Korea) and dissociated using the program “Protein_01”. After the lysates were centrifuged at 4000× *g* for 5 min, the supernatants were collected for protein quantification using Bio-Rad Protein Assay Dye Reagent Concentrate (Cat. 5000006, Bio-Rad Laboratories, Hercules, CA, USA), and the concentration was measured at an OD of 595 nm. Each sample of 30 μg total protein was loaded into each well of 10% sodium dodecyl sulphate-polyacrylamide gel electrophoresis gel and run for electrophoresis. After transferring the proteins to the PVDF (Immobilon^®^-P Transfer Membranes; Cat. 88518, Millipore, Burlington, MA, USA) and being blocked with 5% non-fat dry milk in TBST, the blots were probed overnight at 4 °C with the following primary antibodies (Santa Cruz, Dallas, TX, USA): goat polyclonal antibodies for AT1R (Cat.sc-1173-G, 1:1000 dilution); rabbit polyclonal antibodies for AT2R (Cat.sc-9040, 1:1000 dilution) and MAS1 (Cat. sc-135063, 1:1000 dilution); and mouse monoclonal antibody for AT4R (Cat.sc-365300, 1:1000 dilution) and β-actin (Cat.sc-47778, 1:500,000 dilution). Next, after rinsing with tris-buffered saline with 0.1% Tween 20, the blots were incubated with horseradish peroxidase-coupled donkey anti-Goat IgG (Cat. A15999, Thermo) for AT1R, goat anti-rabbit IgG (Cat. 31460, Thermo) for AT2R and MAS1, and goat anti-mouse IgG (Cat. 31430, Thermo) for AT4R and β-actin at room temperature for 1 h. Finally, the blots were detected using the enhanced chemiluminescence method and quantified by densitometry (Quantity One Analysis software version 4.6.7, Bio-Rad Laboratories) in terms of the integrated optical density. The results were shown as the ratio of AT1R, AT2R, AT4R, and MAS1 over β-actin.

### 4.5. Immunohistochemistry

Immunohistochemical staining was performed for formalin-fixed and paraffin-embedded UAs as previously described [31].The sections were incubated with primary antibodies as used in western blots and the corresponding isotype control at room temperature for 1 h and detected with a secondary antibody using the UltraVision Quanto Detection System HRP DAB Kit (ThermoFisher Scientific Anatomical Pathology, San Diego, CA, USA).

### 4.6. Isometric Tension Measurement

The functions of vasoconstriction and vasodilatation for the fresh UAs were also tested by using a Panlab multi-chamber organ bath system (Panlab Harvard Apparatus, Barcelona, Spain). The dose-dependent vasodilation was tested using papaverine (Sigma, Rockville, MD, USA) under resting tension in the amount of 2 g. After equilibration, the vascular rings were pre-contracted with 10^−9^–10^−5^ M serotonin hydrochloride (5-HT; Sigma). The concentration of 5-HT required to give half of the maximal contraction (EC_50_) was calculated for each individual ring based on the initial concentration–response curve. The rings were then relaxed with 10^−9^–2 × 10^−7^ M papaverine at 37 °C in bubbled (95% O_2_) Krebs solution. At the beginning of the experiment, potassium chloride (100 mM) was used to induce maximal contraction.

### 4.7. Statistics

All data are presented as a median (interquartile range (IQR)) or a number with the proportion of the number. All continuous data between the two groups were analyzed using the Mann–Whitney *U* test, and their relationships with the clinical parameters were analyzed using Spearman’s correlation. Category data were analyzed using a chi-squared test or Fisher’s exact test. Linear regression was performed to determine the independent factor(s) for the perinatal cardiovascular measurements after log or square root transformation for some non-normally distributed variables in the model of GA adjustment. A *p* value < 0.05, determined using SPSS version 20 (SPSS, Inc., Chicago, IL, USA), was considered statistically significant.

## Figures and Tables

**Table 1:** Characteristics and the comparisons of the study population between the normotension and preeclampsia groups.

	Normotension Group (n = 48)	Preeclampsia Group (n = 49)	*p* Value
Maternal or gestational characteristics			
Age (y)	34.13 (30.69–36.23)	34.83 (31.67–38.25)	0.159
BW (kg)	70.00 (63.00–75.00)	76.70 (67.50–86.65)	<0.001 ‡
BH (cm)	160.00 (155.00–163.00)	158.00 (154.50–165.00)	0.763
BMI (kg/m^2^)	26.72 (24.46–29.34)	31.17 (27.02–33.87)	<0.001 ‡
Gravidity	1.50 (1.00–2.00)	2.00 (1.00–3.00)	0.052
Parity	1.00 (1.00–2.00)	2.00 (1.00–2.00)	0.062
SBP (mmHg)	120.50 (111.00–125.00)	167.00 (154.00–185.50)	<0.001 ‡
DBP (mmHg)	73.00 (65.00–79.00)	111.00 (103.00–119.00)	<0.001 ‡
MBP (mmHg)	88.50 (80.00–93.75)	129.00 (120.50–139.50)	<0.001 ‡
Severe PE, n (%)	0 (0.00)	42 (85.71)	N.A.
Essential HTN, n (%)	0 (0.00)	4 (8.16)	N.A.
Proteinuria, n (%)	0 (0.00)	47 (95.92)	N.A.
GA of PE (wk)	N.A.	27.00 (21.50–31.25)	N.A.
Neonatal characteristics			
Male, n (%)	23 (47.92)	19 (38.78)	0.364
GA (wk)	39.14 (38.43–40.14)	33.43 (31.72–35.22)	<0.001 ‡
BBW (g)	3100.00 (2873.75–3307.50)	1500.00 (1080.00–1805.00)	<0.001 ‡
BL (cm)	49.75 (48.00–50.38)	40.00 (36.50–42.50)	<0.001 ‡
BHC (cm)	33.00 (32.00–34.00)	29.00 (26.50–31.00)	<0.001 ‡
SGA, n (%)	0 (0.00)	17 (34.69)	<0.001 ‡
Cesarean section, n (%)	11 (22.92)	46 (93.88)	<0.001 ‡
Apgar score at 1 min	9.00 (9.00–9.00)	8.00 (6.00–9.00)	<0.001 ‡
Apgar score at 5 min	10.00 (10.00–10.00)	10.00 (9.00–10.00)	<0.001 ‡
Neonatal cardiovascular measurements			
HR (bpm)	139.50 (125.00–148.75)	140.00 (123.50–155.50)	0.751
SBP (mmHg)	62.50 (58.00–71.75)	57.50 (50.50–63.00)	0.002 †
DBP (mmHg)	37.50 (31.25–44.75)	29.50 (26.00–34.00)	<0.001 ‡
MBP (mmHg)	46.50 (42.00–53.00)	38.50 (33.00–43.75)	<0.001 ‡
LA (mm)	12.00 (11.00–13.00)	10.70 (9.15–11.90)	<0.001 ‡
AO (mm)	10.00 (9.50–10.88)	8.10 (7.00–9.00)	<0.001 ‡
LVID (mm)	18.55 (17.00–19.60)	15.90 (14.10–17.20)	<0.001 ‡
IVS (mm)	3.28 (3.05–3.53)	2.79 (2.48–3.24)	<0.001 ‡
LVPW (mm)	2.60 (2.50–2.90)	2.08 (1.89–2.39)	<0.001 ‡
LCA (mm)	1.31 (1.18–1.52)	1.31 (1.05–1.55)	0.721
LCA/AO ratio	0.13 (0.11–0.15)	0.16 (0.13–0.19)	<0.001 ‡
RCA (mm)	1.19 (1.01–1.36)	1.10 (0.94–1.33)	0.464
RCA/AO ratio	0.11 (0.10–0.14)	0.15 (0.11–0.18)	<0.001 ‡
FS (%)	30.70 (28.00–34.65)	29.40 (24.70–33.30)	0.285
EF (%)	61.87 (57.17–66.70)	59.61 (52.75–66.20)	0.427
EDV (mL)	10.51 (8.39–12.09)	7.00 (4.87–8.65)	<0.001 ‡
ESV (mL)	3.91 (2.79–5.28)	2.70 (1.84–3.75)	<0.001 ‡
SV (mL)	6.38 (5.55–7.12)	4.04 (2.82–5.34)	<0.001 ‡
CO (L/min)	0.84 (0.72–0.98)	0.55 (0.43–0.70)	<0.001 ‡
CI (L/min/mm^2^)	4.14 (3.39–4.73)	4.14 (3.62–5.76)	0.290

AO = aortic root; CI = cardiac index; CO = cardiac output; BBW = birth body weight; BH = body height; BHC = body head circumference; BL = body length; BMI = body mass index; BW = body weight; DBP, MBP, and SBP = diastolic, mean, and systolic blood pressure, respectively; EDV = end-diastolic volume; EF = ejection fraction; ESV = end systolic volume; FS = fractional shortening; GA = gestational age; HTN = hypertension; HR = heart rate (beats per minute (bpm)); IVS = thickness of interventricular septum; LA = left atrium; LCA = left main coronary artery; LVID = inner diameter of left ventricle; LVPW = thickness of left ventricular posterior wall; N.A. = not applicable; RCA = right coronary artery; SGA = small GA (defined as BBW lower than the tenth percentile); SV = stroke volume. † *p* < 0.01. ‡ *p* < 0.001. Compared using Mann–Whitey *U* test between groups.

**Table 2:** Multivariable analysis for cell- or tissue-specific independent factor(s) of perinatal cardiovascular features under stepwise linear regression in the GA-adjusted models, performed individually for the PE and normotension groups.

PE					Normotension				
	Coefficient	S.E.	*p*	Adjusted R^2^		Coefficient	S.E.	*p*	Adjusted R^2^
SBP					SBP				
Constant	65.17	3.11	<0.001 ‡	21.97%	Constant	−89.35	65.60	0.187	16.00%
GA			0.73		GA	3.88	1.67	0.030 *	
Lg *ANPEP/S18* (CBL)	19.02	6.96	0.012 *		SQRT cGMP (UA)			0.071	
Constant	41.76	4.49	<0.001 ‡	30.05%					
GA			0.268						
SQRT cAMP (UA)	12.20	3.85	0.005 †						
Lg DBP					Lg DBP				
Constant	1.58	0.04	<0.001 ‡	43.51%	Constant	2.09	0.20	<0.001 ‡	35.65%
GA			0.264		GA			0.944	
Lg *AT2R/GAPDH* (UA)	−0.33	0.11	0.012 *		AT2R (UA, WB)	−0.33	0.13	0.024 *	
Lg *ACE2/GAPDH* (UA)			0.751		Constant	1.80	0.07	<0.001 ‡	29.81%
					GA			0.415	
					SQRT cAMP (UA)			0.732	
					SQRT cGMP (UA)	−0.17	0.05	0.003 †	
LVID					LVID				
Constant	−1.48	0.69	0.053	55.67%	Constant	0.78	0.47	0.132	31.64%
GA	0.07	0.02	0.003 †		GA			0.222	
*AT4R/GAPDH* (CP-EC)	0.62	0.26	0.033 *		AT2R (UA, WB)	0.72	0.29	0.033 *	
IVS					IVS				
N.A.					Constant	0.20	0.05	0.001 †	26.55%
					GA			0.602	
					*AT4R/GAPDH* (CP-EC)	0.12	0.05	0.024 *	
LVPW					LVPW				
Constant	0.36	0.05	<0.001 ‡	27.89%	Constant	0.21	0.03	<0.001 ‡	15.24%
GA			0.749		GA			0.208	
*AT4R/GAPDH* (CP-EC)	−0.12	0.05	0.025 *		SQRT cAMP (UA)			0.462	
					SQRT cGMP (UA)	0.04	0.02	0.034 *	
AO					AO				
Constant	−7.81	2.29	0.003 †	69.30%	Constant	9.81	0.20	<0.001 ‡	34.8%
GA	0.47	0.07	<0.001 ‡		GA			0.117	
SQRT cAMP (UA)			0.120		Lg *ACE/GAPDH* (UA)	−2.60	0.99	0.026 *	
SQRT cGMP (UA)			0.335						
LCA/AO					LCA/AO				
Constant	0.06	0.03	0.072	38.24%	N.A.				
GA			0.709						
APN (CP)	0.01	0.003	0.002 †						
Lg RCA/AO					Lg RCA/AO				
Constant	−1.10	0.10	<0.001 ‡	18.51%	N.A.				
GA			0.828						
APN (CP)	0.02	0.01	0.029 *						
Lg EDV					Lg EDV				
N.A.					Constant	0.43	0.28	0.148	28.14%
					GA			0.255	
					AT2R (UA, WB)	0.39	0.17	0.044 *	
					Constant	1.25	0.11	<0.001 ‡	20.09%
					GA			0.601	
					*ACE/GAPDH* (CP-EC)	−0.22	0.10	0.046 *	
					Constant	1.08	0.02	<0.001 ‡	51.55%
					GA			0.601	
					Lg *ACE/GAPDH* (UA)	−0.41	0.12	0.005 †	
ESV					ESV				
Constant	0.30	1.02	0.776	18.32%	Constant	5.28	0.35	<0.001 ‡	41.32%
GA			0.150		GA			0.525	
APN (CP)	0.22	0.10	0.034 *		Lg *ACE/GAPDH* (UA)	−5.16	1.74	0.014 *	
EF					EF				
Constant	50.17	4.78	<0.001 ‡	16.34%	N.A.				
GA			0.886						
SQRT cGMP (UA)	9.59	4.33	0.039 *						
Lg SV					Lg SV				
Constant	−1.38	0.37	0.001 †	64.06%	Constant	0.67	0.06	<0.001 ‡	28.02%
GA	0.05	0.01	<0.001 ‡		GA			0.972	
SQRT cAMP (UA)	0.18	0.07	0.023 *		SQRT cAMP (UA)			0.873	
SQRT cGMP (UA)			0.066		SQRT cGMP (UA)	0.14	0.04	0.005 †	
					Constant	0.91	0.05	<0.001 ‡	16.21%
					GA			0.344	
					APN (CP)	−0.01	0.006	0.036 *	
					Constant	0.17	0.26	0.534	34.99%
					GA			0.104	
					AT2R (UA, WB)	0.42	0.16	0.025 *	
SQRT CO					SQRT CO				
Constant	−0.97	0.46	0.055	50.25%	Constant	−0.12	0.33	0.726	47.28%
GA	0.03	0.01	0.016 *		GA			0.157	
*AT4R/GAPDH* (CP-EC)	0.48	0.17	0.016 *		AT2R (UA, WB)	0.67	0.20	0.008 †	
CI					CI				
Constant	−4.33	3.00	0.170	32.80%	Constant	23.21	13.16	0.112	64.46%
GA			0.724		GA	−0.72	0.31	0.047 *	
Lg Ang1–7 (MP)	3.05	1.06	0.012 *		AT2R (UA, WB)	6.02	1.73	0.007 †	
Constant	3.20	0.90	0.001 †	14.79%	Constant	2.40	1.10	0.040 *	13.48%
GA			0.763		GA			0.464	
SQRT cGMP (UA)	1.72	0.81	0.048 *		SQRT cGMP (UA)	1.64	0.78	0.048 *	
					Constant	4.75	0.42	<0.001 ‡	35.32%
					GA			0.339	
					Lg *ACE/GAPDH* (UA)	−5.65	2.13	0.024 *	
SBP (maternal)					SBP (maternal)				
Constant	161.47	4.38	<0.001 ‡	43.99%	Constant	115.69	1.39	<0.001 ‡	80.63%
GA			0.713		GA			0.741	
Lg *AT1R/GAPDH* (UA)	48.99	15.78	0.011 *		Lg *AT1R/GAPDH* (UA)	−24.70	3.78	<0.001 ‡	
Constant	127.86	17.47	<0.001 ‡	32.26%	Lg *AT2R/GAPDH* (UA)			0.476	
GA			0.343		Lg *ACE/GAPDH* (UA)			0.260	
*AT1R/GAPDH* (CP-EC)	29.91	10.48	0.013 *		Lg *ACE2/GAPDH* (UA) Lg *MAS1/GAPDH* (UA)			0.670 0.144	
Lg DBP (maternal)					Lg DBP (maternal)				
Constant	2.01	0.01	<0.001 ‡	38.81%	Constant	1.86	0.01	<0.001 ‡	26.72%
GA			0.172		GA			0.377	
Lg *AT1R/GAPDH* (UA)	0.13	0.05	0.020 *		Lg *MAS1/GAPDH* (UA)	−0.11	0.05	0.049 *	

ACE2 = angiotensin converting enzyme-2; Ang1–7 = angiotensin 1–7; ANPEP = alanyl aminopeptidase (membrane); AO = aortic root; APN = aminopeptidase-N; AT1R = angiotensin II receptor 1; AT2R = angiotensin II receptor 2; AT4R = angiotensin II receptor 4; CI = cardiac index; CO = cardiac output; CP = cord blood plasma; CP-EC = CP-stimulated endothelial cell; DBP = diastolic blood pressure; EDV = end diastolic volume; ESV = end systolic volume; GA = gestational age; HUAEC = human umbilical arterial endothelial cell; LA = left atrium; LCA = left main coronary artery; Lg = log; LVID = inner diameter of left ventricle; LVPW = posterior wall of left ventricle; MP = maternal plasma; N.A. = not applicable; SBP = systolic blood pressure; SQRT = square root; SV = stroke volume; UA = umbilical artery; WB = western blot. * *p* < 0.05. † *p* < 0.01. ‡ *p* < 0.001.

## Data Availability

The corresponding author had full access to all data in the study and takes responsibility for its integrity and the data analysis. All data generated or analyzed during this study are included in this article. Further inquiries can be directed to the corresponding author.

## Supplementary Materials

The supporting information can be downloaded at https://www.mdpi.com/article/10.3390/ijms25137426/s1.
