# Supplementary material for: Association of Perinatal Cardiovascular Features with Angiotensin System Expressions in Maternal Preeclampsia
Source: Int J Mol Sci. 2024 Jul 6;25(13):7426. doi: 10.3390/ijms25137426 (PMC11242154; doi:10.3390/ijms25137426)
Supplement: Supplementary file 1 [file ijms-25-07426-s001.zip › ijms-3054962-supplementary.pdf]

Supplementary Table S1. The comparisons of angiotensin-related molecules between the normotension and preeclampsia groups.

	Normotension Group	Preeclampsia Group	*P* value
Plasma			
NEP, pg/ml (MP)	180.23 (75.08–314.93)	423.13 (93.96–1228.60)	*0.131*
NEP, pg/ml (CP)	116.06 (68.91–230.86)	113.40 (53.87–158.27)	*0.469*
Maternal blood leukocytes			
AT1R/18S	1.19 (0.23–4.47)	0.56 (0.24–5.63)	*0.975*
AT2R/18S	1.26 (0.19–4.59)	0.75 (0.23–2.90)	*0.821*
AT4R/18S	0.93 (0.70–1.53)	0.94 (0.67–1.25)	*0.726*
MAS1/18S	0.89 (0.37–4.34)	1.03 (0.16–3.34)	*0.853*
ACE/18S	1.30 (0.64–1.78)	1.22 (0.86–2.22)	*0.680*
ACE2/18S	1.00 (0.25–3.41)	0.92 (0.24–2.75)	*0.869*
ANPEP/18S	1.09 (0.72–1.41)	0.75 (0.48–1.26)	*0.115*
MME/18S	1.10 (0.55–1.70)	0.80 (0.46–1.59)	*0.208*
Cord blood leukocytes			
AT1R/18S	0.70 (0.33–3.69)	1.66 (0.34–5.37)	*0.601*
AT2R/18S	0.92 (0.30–3.63)	0.80 (0.38–4.87)	*0.782*
AT4R/18S	0.86 (0.78–1.45)	1.02 (0.87–1.49)	*0.382*
MAS1/18S	0.84 (0.46–2.21)	0.97 (0.46–3.74)	*0.469*
ACE/18S	0.97 (0.71–1.59)	1.09 (0.81–1.82)	*0.449*
ACE2/18S	0.80 (0.34–3.31)	0.90 (0.45–4.42)	*0.773*

ACE, angiotensin-converting-enzyme; ACE2, angiotensin-converting-enzyme 2; ANPEP, alanyl aminopeptidase, membrane; APN, aminopeptidase-N; AT1R, angiotensin II type 1 receptor; AT2R, angiotensin II type 2 receptor; AT4R, angiotensin II type 4 receptor; MME, membrane metalloendopeptidase; NEP, neprilysin. Comparisons by Mann-Whitey *U* test between groups.

Supplementary Table S2. The sequences of primer pairs of intended genes and house-keeping genes.

Gene	Gene description	Primer sequence
18S	RNA, *18S* ribosomal	Forward: GTAACCCGTTGAACCCCATT
		Reverse: CCATCCAATCGGTAGTAGCG
ACE	angiotensin I converting	Forward: CGGCTCAATGGCTATGTAGATG
	enzyme 1	Reverse: CAGGTTGAGGTAGAGTGGCT
ACE2	angiotensin I converting	Forward: CATGCTAACGGACCCAGGAA
	enzyme 2	Reverse: TAAGGATCCTGAAGTCGCCC
ANPEP	alanyl aminopeptidase,	Forward: ACGGAGTTCCAGAGTGTGAG
	membrane (aminopeptidase N)	Reverse: GCGATAGCGTTGCAGTAGAC
ATR1	angiotensin II receptor, type 1	Forward: CAGATGACGGCTGCTCGAAG
		Reverse: TGGAAACTGGACAGAACAATCTGG
ATR2	angiotensin II receptor, type 2	Forward: TATGGCCTGTTTGTCCTCAT
		Reverse: CATTGGGCATATTTCTCAGG
AT4R	placental leucine	Forward: CAGCCTTCAGATACAAGCTACC
(LNPEP)	aminopeptidase	Reverse: TGACCCACAGCACTTCTTCT
GAPDH	glyceraldehyde-3-phosphate	Forward: AATTCCATGGCACCGTCAAG
	dehydrogenase	Reverse: ATCTCGCTCCTGGAAGATGG
MAS1	MAS1 proto-oncogene	Forward: TGACGGCCATTAGTGTGGAG
		Reverse: CAGACCAATGCCGACTGGTA
MME	membrane metallo-	Forward: ATGGAGACCTCGTTGACTGG
	endopeptidase (neprilysin)	Reverse: CATTAAGGTGCTGTCCACCTG
VCAM1	vascular cell adhesion	Forward: CCGAAAGGCCCAGTTGAAG
	molecule 1	Reverse: AGCACGAGAAGCTCAGGAGAA
